# Magnetic drug-loaded microbubbles for treating lower limb venous thrombosis under controllable rotating magnetic field

**DOI:** 10.3389/fbioe.2025.1615863

**Published:** 2025-11-12

**Authors:** Yu-Ming Huang, Cheng-Rang Liu, Yi-Qi Xu, Chao Cao, Zhen-Gan Huang, Hong-Wen Fei, Yue-Shan Huang

**Affiliations:** 1 School of Medicine, South China University of Technology, Guangzhou, China; 2 Department of Catheterization Lab, Guangdong Cardiovascular Institute, Guangdong Provincial Key Laboratory of South China Structural Heart Disease, Guangdong Provincial People’s Hospital (Guangdong Academy of Medical Sciences), Southern Medical University, Guangzhou, China; 3 School of Materials Science and Engineering, South China University of Technology, Guangzhou, Guangdong, China; 4 Department of Pediatrics, Nanfang Hospital, Southern Medical University, Guangzhou, China; 5 Guangdong Cardiovascular Institute, Guangdong Provincial People’s Hospital (Guangdong Academy of Medical Sciences), Southern Medical University, Guangzhou, China

**Keywords:** poly (lactic-co-glycolic acid) microbubbles, magnetic drug delivery, rotating magnetic field, thrombolysis, single-chain urokinase-type plasminogen activator (proUK), acute lower limb venous thrombosis

## Abstract

**Objective:**

This study aimed to develop a poly (lactic-co-glycolic acid) (PLGA)-based magnetic loaded iron oxide (Fe_3_O_4_) and single-chain urokinase-type plasminogen activator (proUK) for enhancing thrombolysis under a controlled rotating magnetic field, specifically targeting acute lower limb venous thrombosis.

**Background:**

Acute thrombotic disorders are significant health threats, however, the exploration of magnetic actuation as a treatment for acute thrombosis has been limited.

**Methods:**

Magnetic microbubbles were prepared using a double emulsion method, loaded with Fe_3_O_4_ nanoparticles and proUK. The microbubble characteristics were analyzed through chemical, physical, and biological related technologies.

**Results:**

Fe_3_O_4_ nanoparticle loading was confirmed by X-ray diffraction, and the encapsulation efficiency of the magnetic microbubbles was determined using an ELISA kit and colorimetric assay, reaching a maximum of 56.65% at a proUK concentration of 7.5 mg/mL. Thrombolysis efficiency was significantly enhanced under a rotating magnetic field of 1.5 mT and 6 Hz frequency, achieving up to 25% lysis rate *in vitro*, markedly higher than control conditions. Furthermore, *in vivo* experiments using a rabbit model of hindlimb venous thrombosis validated the efficacy of this approach, with Color Doppler Flow Imaging showing restored blood flow and elevated D-dimer levels indicating effective thrombus dissolution.

**Conclusion:**

This novel magnetic drug delivery system, combined with a rotating magnetic field, demonstrates excellent thrombolysis efficiency and presents a promising and safe therapeutic strategy for acute venous thrombosis.

## 1 Introduction

In developed countries, thrombotic diseases are a major cause of death and disability (Sogaard et al., 2014), but early diagnosis and treatment of thrombosis can effectively reduce morbidity and mortality (Wells et al., 2014; Kearon et al., 2016). Thrombolysis through intravenous administration of plasminogen activators such as urokinase is a clinical therapeutic approach to reduce thrombus formation and embolism (Hacke et al., 2008; Caplan, 2006). High-dose administration can enhance the thrombolytic effect, but it may also lead to serious consequences such as bleeding (Caplan, 2006). Therefore, the goal of optimal efficacy, efficiency, and safety has not yet been maximized. How to find a thrombolytic method with better efficacy and higher safety is a hot topic in clinical thrombolytic therapy.

Magnetic nanoparticles offer several advantages when used as drug carriers, including a large surface area that can be appropriately modified to bind with drug molecules (Arias et al., 2018; Lee and Park, 2023; Dadfar et al., 2019; Gao et al., 2016). To ensure biocompatibility and non-toxicity, iron oxide-based particles (magnetite) with superparamagnetic characteristics are commonly used as the magnetic responsive component, which can be manipulated through an external magnetic field gradient (Mahmoudi et al., 2011; Xu and Sun, 2013; Cheng et al., 2014). Superparamagnetic behavior indicates that their magnetization disappears once the external magnetic field is removed (Gupta et al., 2007). Based on these properties, superparamagnetic nanoparticles can be transported through the vascular system and, under the guidance of a magnetic field, can rapidly release drugs at specific sites, thereby improving treatment efficiency.

A substantial body of research has demonstrated that ultrasound irradiation can enhance the effectiveness of thrombolytics, and the introduction of ultrasound contrast microbubbles can further improve this effect due to the acceleration of cavitation enhancement by the interaction between ultrasound radiation and microbubbles (Kim et al., 2017). However, safety is another critical issue because the biophysical effects induced by the combination of ultrasound irradiation and microbubbles have been widely reported to affect adjacent tissues, such as vascular endothelium (Huang et al., 2017; Nakamura et al., 2016; Chen et al., 2012). The frequency for ultrasound thrombolysis is typically above 1 MHz, and high energy levels can produce other side effects on other tissues within the body (Chen et al., 2019; Hoogenboom et al., 2015). In contrast, using low-energy magnetic field-driven microbubble particles, the magnetic field lines do not harm tissues and will not cause secondary damage to tissues due to high mechanical energy.

## 2 Materials and methods

### 2.1 Materials

The poly (lactic-co-glycolic acid) copolymer (PLGA) with a molecular weight of 15,000 (containing 75% lactide and 25% glycolide) used in this study was purchased from Jinan Daigang Bio-Engineering Co., Ltd. (Shandong Province, China). Additionally, we procured dichloromethane (DCM), polyvinyl chloride (PVC), and iron oxide nanoparticles surface-modified with oleic acid (Fe_3_O_4_) from Aladdin Co., Ltd. (China). The single-chain urokinase-type plasminogen activator (proUK) was provided by Tasly Biopharm Co., Ltd. The phosphate buffer saline (PBS) was sourced from Xiamen Haibiao Technology Co., Ltd. For experimental purposes, we purchased human umbilical vein endothelial cells (HUVECs) from Wuhan Promega Life Science and Technology Co., Ltd. Meanwhile, the fetal bovine serum and endothelial cell medium (ECM) required for the experiment were both purchased from Thermo Fisher Scientific Biochemicals (China) Ltd. The cell culture plates were provided by Corning Costar, while the Cell Counting Kit-8 reagent was sourced from Dojindo Laboratories in Japan. Finally, the enzyme-linked immunosorbnent assay (ELISA) kit used to detect single-chain urokinase-type plasminogen activator (proUK) was purchased from Jingmei Biotechnology Co., Ltd. (Jiangsu, China).

### 2.2 Preparation magnetic microbubbles

The preparation of magnetic rotation microbubbles (MRB) employed a modified double emulsion method reported in the literature (Sun et al., 2016; Jiang et al., 2022; Zhang et al., 2013). Specifically, PLGA (50 mg/mL) and Fe_3_O_4_ (0.5 mg/mL) were fully dissolved in 2 mL of dichloromethane to form the organic phase (O). Subsequently, 0.4 mL of double distilled water was added as the inner water phase (W1), and ultrasonic oscillation (with a power of 100 w) was applied for 50 s to prepare the primary emulsion (W1/O). Then, 10 mL of 4% polyvinyl alcohol, vinylalcohol polymer (PVA) solution (serving as the outer water phase, W2) was poured in, and a high-speed homogenizing disperser was used at a speed of 8,000 rpm/min for 5 min of homogenization to generate a double emulsion (W1/O/W2). Afterwards, 10 mL of 2% isopropanol solution was added, and the mixture was continuously stirred for 2 h at room temperature until the surface of the microbubbles solidified and the dichloromethane completely evaporated. Finally, the mixture was centrifuged using a high-speed frozen centrifuge, washed with double distilled water, and the supernatant was removed. The obtained magnetic rotation microbubbles (MRB) were collected, freeze-dried, and stored for future use.

The process of preparing Fe_3_O_4_@PLGA@proUK (MRB@proUK) microbubbles is similar to that of preparing MRB, but with a key difference: we used a proUK solution (concentration of 3 mg/mL) as the inner water phase instead of 0.4 mL of double distilled water. Additionally, when preparing microbubbles containing proUK, special measures were taken to maintain the activity of proUK by placing the samples in an ice bath to prevent the deactivation of proUK. The preparation of the magnetically rotating drug-loaded microbubbles was carried out at room temperature (20 °C), and no substances that could inactivate proUK were added during the process. Therefore, the activity of proUK was not affected during the preparation.

### 2.3 Preparation thrombus

The preparation method of blood clot referred to the procedure described in the reference (Zhang et al., 2019). Specifically, freshly collected rabbit blood was mixed with 5 wt% CaCl_2_ solution at a volume ratio of 25:1, which means 1 mL of CaCl_2_ solution was added to every 25 mL of rabbit blood. Subsequently, the mixed blood solution was injected into eppendorf (EP) tubes. Then, the tubes containing blood were incubated in a 37 °C constant temperature water bath for 4 h. To ensure that the blood clot does not spontaneously degrade, we store it at 5 °C before use.

### 2.4 Preparation controllable rotating magnetic field

**FIGURE 1:**
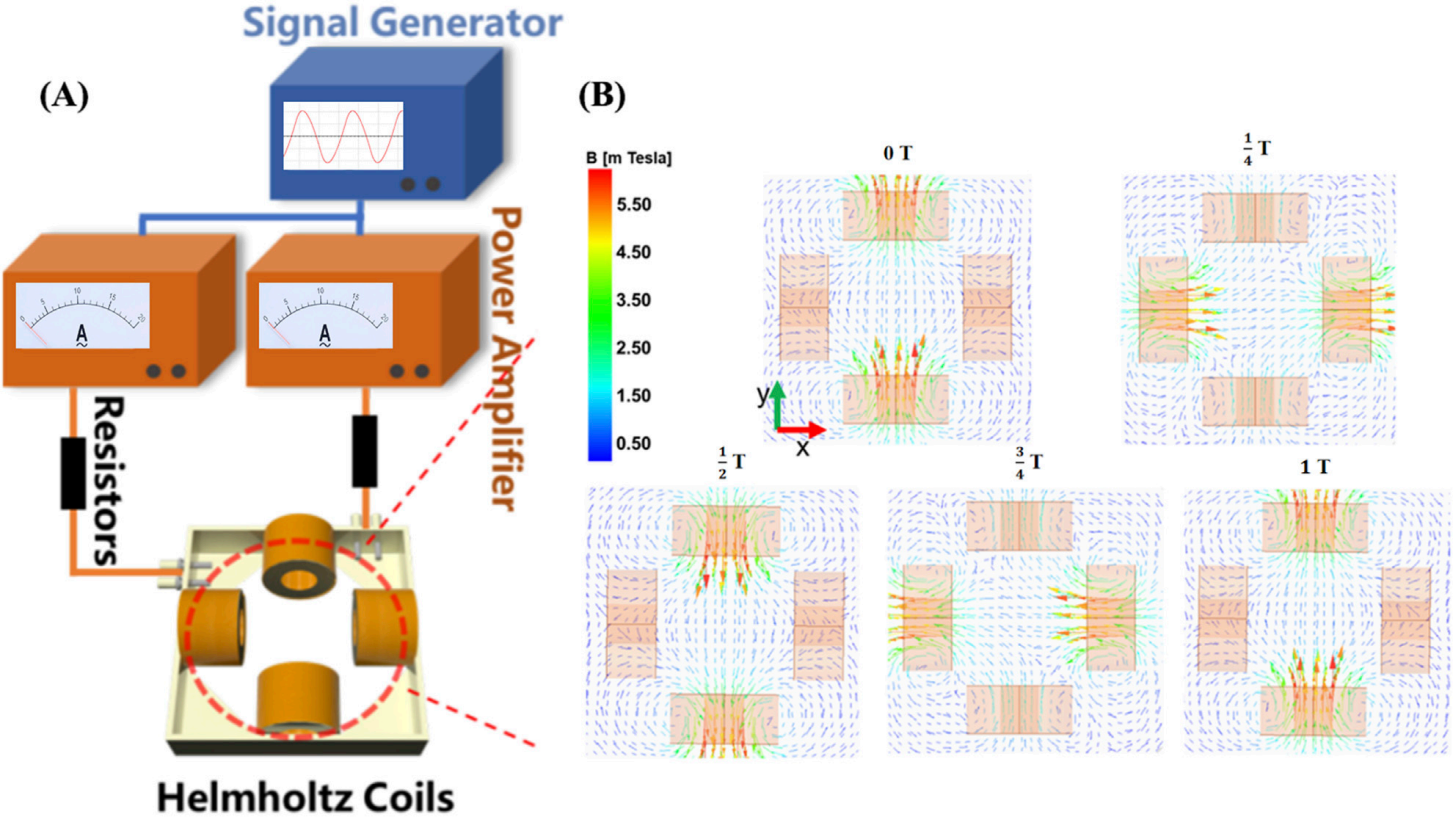
Rotating magnetic field generator. **(A)** A controllable magnetic rotation device composed of a signal generator, power amplifier, resistor, and Helmholtz coil; **(B)** Simulation of magnetic field lines rotating in a periodic manner.

The rotating magnetic field is generated through two pairs of Helmholtz coils, which are mounted on a 3D-printed square frame, and equipped with resistors (ER), a signal generator (SG), and a power amplifier (PA). By adjusting the amplitude and frequency of the signal generator, we can generate an alternating magnetic field with varying magnetic field strengths (B = 0–2 mT) and frequencies (f = 0–8 Hz). Compared to the magnetic field generated by permanent magnets, the magnetic field generated by this device exhibits significant advantages in terms of flux density uniformity, frequency controllability, adjustable magnetic field strength, and controllable magnetic field waveform. As shown in Figure 1A, the central area of the coils is set as the application region. To gain a deeper understanding of the physical mechanisms of the rotating magnetic field, we simulated the magnetic field generated by the coils using Maxwell software. Figure 1B illustrates the simulated magnetic flux density distribution of the magnetic field on the XY plane. The arrow directions in the figure indicate the standard directions of magnetic flux density. Through the simulation results in Figure 1, we can clearly observe the distribution of magnetic field strength within the working area of the magnetic rotation device and the variations in magnetic induction lines over a complete cycle, indicating that magnetic microbubbles can freely rotate within this magnetic field.

### 2.5 Microbubble composition characterization

A minimal amount of microbubbles was uniformly pressed into the sample holder and characterized using an X-ray diffractometer (Empyrean, PANalytical, Netherlands). Cu Kα radiation was employed as the X-ray source, and the measurement was conducted at room temperature. The scanning rate was set at 5°/min with a 2θ range from 10° to 80°. The X-ray diffraction (XRD) patterns obtained were compared against a standard database to achieve precise interpretation of the sample’s crystalline characteristics.

Microbubbles prepared under different conditions were selected for thermogravimetric (TG) analysis. The testing conditions were set as follows: samples were heated from 30 °C to 800 °C at a rate of 10 K/min in an oxygen atmosphere. By comparing the weight loss rates of microbubbles prepared with different ratios of Fe_3_O_4_ to PLGA, the magnetic particle content in each sample was quantitatively analyzed. This method aids in evaluating the thermal stability and compositional characteristics of the microbubbles at high temperatures.

### 2.6 The size, zeta potential and morphological characteristics of microbubbles

After dissolving the appropriate microbubble in double-distilled water, we conducted detailed characterization using various advanced microscopy techniques. Specifically, we comprehensively observed the external morphology using an Olympus CKX41 optical microscope (provided by Olympus Co. Ltd., Tokyo, Japan) and a MERLIN scanning electron microscope from Carl Zeiss in Germany. Meanwhile, to further explore its internal structure, we utilized a Talos L120c transmission electron microscope (provided by Thermo Fisher). Using the Leica TCS-SP2 laser scanning confocal microscope (produced by Leica Microsystems, Wetzlar, Germany), we successfully confirmed the application of proUK on microbubbles (MB), where proUK was labeled with fluorescein isothiocyanate (FITC) for tracking purposes. Additionally, at a constant temperature of 25 °C, we accurately measured the size and zeta potential of different microbubbles using a Zetasizer Nano ZS90 laser particle size analyzer (manufactured by Malvern Instruments Ltd, Worcs, United Kingdom). To evaluate the Fe_3_O_4_ content in MRB@proUK, we adopted inductively coupled plasma-optical emission spectrometry (ICP-OES). The concentration of proUK in the supernatant was determined using an ELISA kit and colorimetry at a wavelength of 450 nm. Furthermore, we calculated the encapsulation efficiency using the formula: “Encapsulation efficiency of proUK (%) = (Amount of proUK added - Amount of proUK in the supernatant)/(Total amount of proUK added) × 100%”. Finally, a hysteresis loop test was conducted using Quantum Design PPMS Dynacool equipment to measure the saturation magnetization value of MRB@proUK microbubbles.

### 2.7 Study on the *in Vitro* cytocompatibility of microbubbles

HUVEC cells were cultured in a complete medium containing 10% fetal bovine serum and 90% ECM, and experiments were conducted between cell passages 3 to 8. To assess cell proliferation, we used the Cell Counting Kit-8 (CCK-8) for measurement, following the method described in reference (Yang et al., 2021). The experimental steps are as follows: Firstly, different microbubble samples with a concentration of 2107 MRBs/mL were placed in a 96-well plate, with 2000 cells evenly seeded into each well, and cultured for 1, 3, and 5 days, respectively. Subsequently, CCK-8 was added to each well and incubated for 1 h in a constant temperature dark environment at 37 °C. Finally, the supernatant after incubation was transferred to a new 96-well plate, and the optical density (OD) value at a wavelength of 450 nm was measured using a microplate reader. In addition, we cultured the cells for 1, 3, and 5 days using the aforementioned method and stained them with a 200 μM homemade staining solution consisting of Calcein acetoxymethyl ester (AM) and propidium iodide (PI) at a ratio of Calcein AM:PI = 1:2, where the ratio of Calcein AM to PBS was 1:1000. After incubating in dark conditions for 30 min, images of live cells (labeled with green fluorescence by Calcein-AM) and dead cells (labeled with red fluorescence by propidium iodide) were captured using an inverted fluorescence microscope for further analysis of cell viability, following the method described in reference (Kim et al., 2018).

### 2.8 Study on the use of microbubbles in magnetic rotation thrombolysis *in Vitro*

**FIGURE 2:**
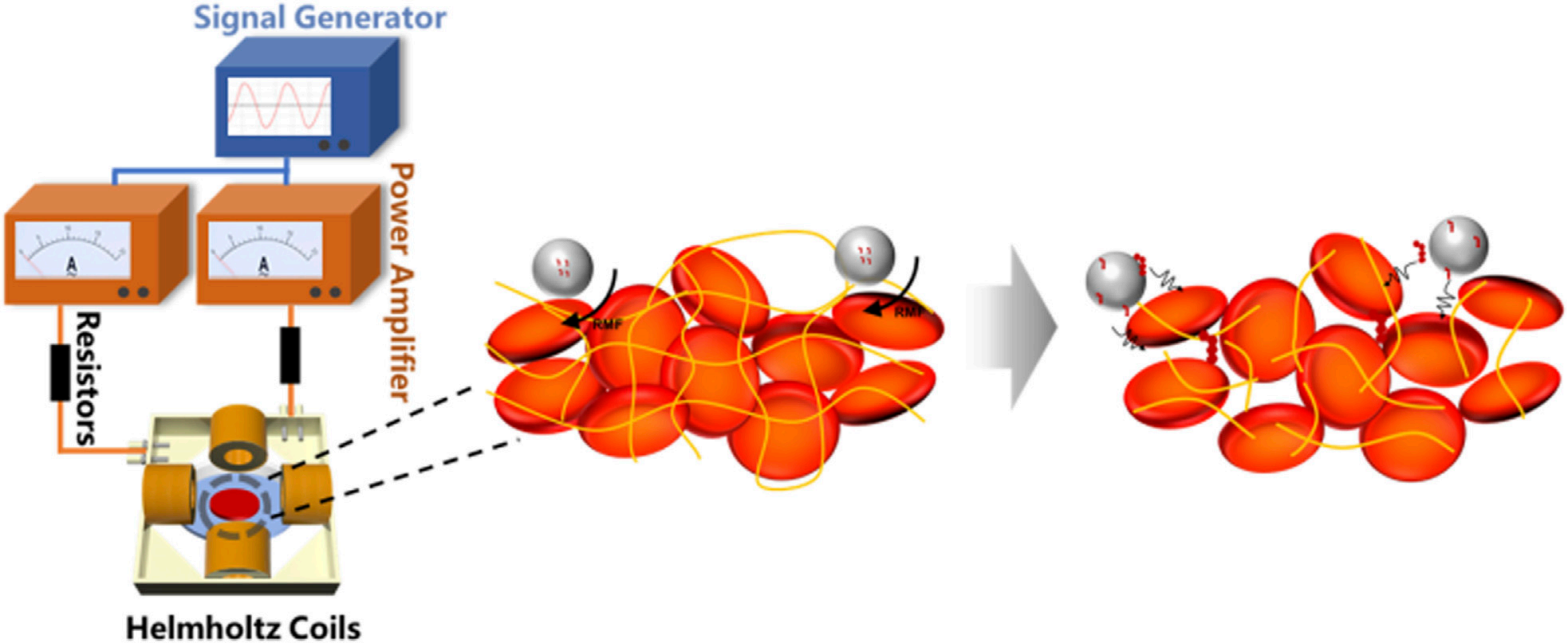
*In vitro* thrombolysis under the rotating magnetic field.

As shown in Figure 2, during each experiment, a clot sample with a mass of 300 mg ± 10% was placed within the working area at the center of the coil. The petri dish was filled with normal saline and maintained at a constant temperature of 37.0 °C ± 0.3 °C. A signal generator was utilized to control the magnetic field strength and frequency within the central region. The thrombolysis rate was calculated by monitoring the change in mass of the blood clot, with specific calculation methods referenced from the literature (Zhou et al., 2014). The formula for calculating the thrombolysis rate is:

$$\text{thrombolysis rate}=\frac{weight\;reduction\;of\;the\;clot}{weight\;of\;the\;primary\;clot}\;x\;100\%$$

### 2.9 Statistical analysis

In this study, the continuous variables in this study were presented using the mean accompanied by the standard deviation. In cases where variables followed a normal distribution, t-tests coupled with *post hoc* analysis were utilized for comparisons. Variables that did not conform to a normal distribution were analyzed using Mann-Whitney U tests in conjunction with *post hoc* analysis, as well as one-way analysis of variance. The categorical variables in this study were represented in terms of their frequencies. Comparisons of categorical variables were conducted using Chi-square tests and Fisher’s exact tests. Comparisons of categorical variables were conducted using Chi-square tests and Fisher’s exact test. All statistical analyses in this study were performed using MedCalc software (version 14.12, MedCalc Software, Ostend, Belgium). In the statistical results, “NS” denotes no statistical significance, while *P < 0.05, **P < 0.01, and ***P < 0.001 indicate levels of statistical significance, respectively.

## 3 Result

### 3.1 Microbubble composition

**FIGURE 3:**
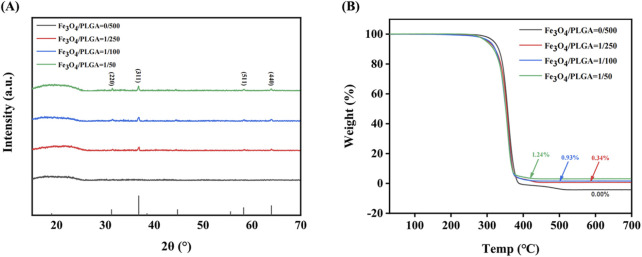
Characterization of microbubble composition. **(A)** The XRD pattern of Fe_3_O_4_/PLGA microbubbles; **(B)** The TGA chart of Fe_3_O_4_/PLGA. XRD: X-ray diffraction, PLGA: poly (lactic-co-glycolic acid), TGA: thermogravimetric analysis.

The results of X-ray diffraction (Figure 3A) reveal the crystalline structure information of Fe_3_O_4_/PLGA magnetic microbubbles. By analyzing the diffraction peaks at different 2θ angles, it can be confirmed that the characteristic peaks of Fe_3_O_4_ crystals appear at approximately 31°, 36°, 59°, and 65°, corresponding to the (220), (311), (511), and (440) crystal planes of the Fe_3_O_4_ phase. The presence of these characteristic peaks indicates the absence of other impurity phases, confirming the purity of the magnetite phase in the sample. The intensity of the diffraction peaks is proportional to the content of Fe_3_O_4_ nanoparticles in the sample; higher crystalline content leads to stronger diffraction peaks. Additionally, the sharpness of the diffraction peaks is an important indicator of crystal quality; sharper peaks indicate fewer lattice defects, which is advantageous for magnetic applications as it ensures uniformity and strong magnetic response. In contrast, the XRD pattern of blank PLGA microbubbles does not show any distinct sharp peaks, indicating that the material is amorphous, a common characteristic of many polymeric materials.

The thermogravimetric analysis (TGA) curve (Figure 3B) shows the mass change of magnetic microbubbles when heated from 30 °C to 800 °C at a rate of 10 K/min in an oxygen atmosphere. This data validates the thermal stability and decomposition characteristics of the microbubbles during heating. Within the temperature range of 30 °C–350 °C, the mass of the microbubbles remains stable, indicating no significant loss of volatile substances within this interval. This suggests that the microbubbles contain either no or only trace amounts of volatile components, such as residual solvents or adsorbed water. When the temperature rises to around 350 °C, there is a sharp decrease in the mass of all samples, marking the onset of PLGA decomposition. As a biodegradable polymer, PLGA undergoes ester bond cleavage at this temperature through hydrolysis or oxidation reactions, forming smaller molecular fragments such as lactic acid and glycolic acid, which subsequently escape from the system, leading to mass reduction. Additionally, as the Fe_3_O_4_ content increases, the amount of solid residue remaining after thermal decomposition also increases, reflecting the thermal stability of Fe_3_O_4_. Since Fe_3_O_4_ is an inorganic compound, it does not decompose like PLGA during the thermal process, thus the remaining solid at 800 °C is primarily Fe_3_O_4_.

**FIGURE 4:**
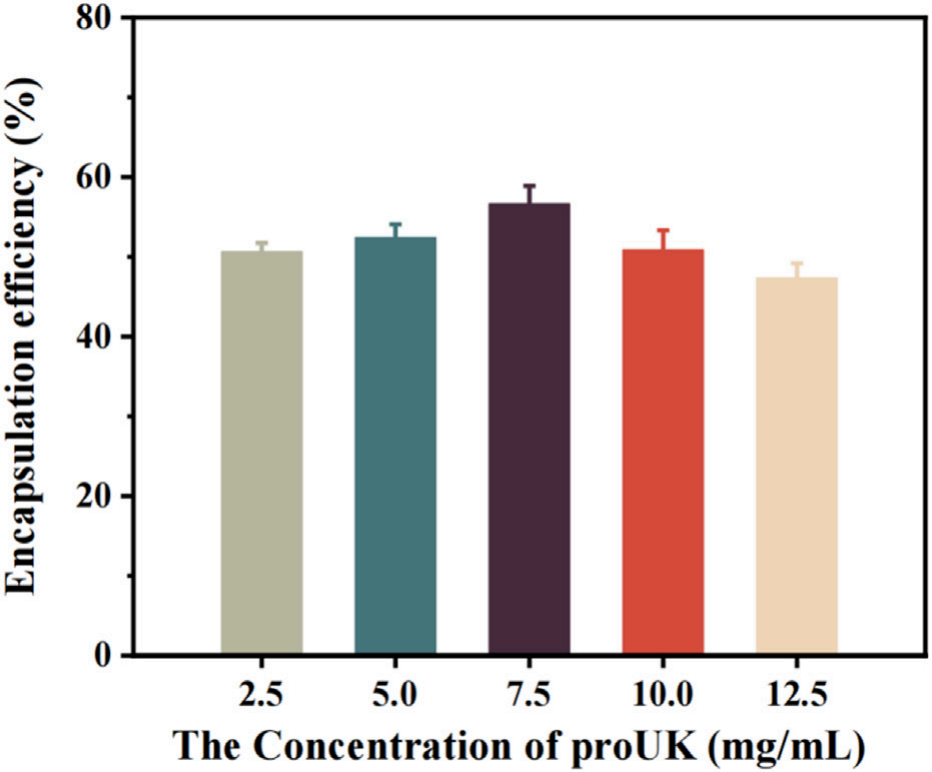
The relationship between the encapsulation efficiency of magnetic spin loaded drug microbubbles and drug concentration.

Figure 4 illustrates the relationship between the drug concentration and the encapsulation efficiency of magnetically rotating drug-loaded microbubbles. When the loaded drug concentration is below 7.5 mg/mL, the encapsulation efficiency increases with increasing drug concentration, reaching a maximum of 56.65%. This may be attributed to the availability of more binding sites on the microbubble surface at lower drug concentrations, allowing for greater incorporation of drug molecules. The increasing encapsulation efficiency suggests that the microbubbles exhibit strong drug-encapsulation capability during this phase.However, when the drug concentration exceeds 7.5 mg/mL, the encapsulation efficiency begins to decline. This phenomenon may be related to the saturation of binding sites on the microbubble surface—beyond this threshold, the microbubbles are no longer able to effectively encapsulate additional drug molecules.

**FIGURE 5:**
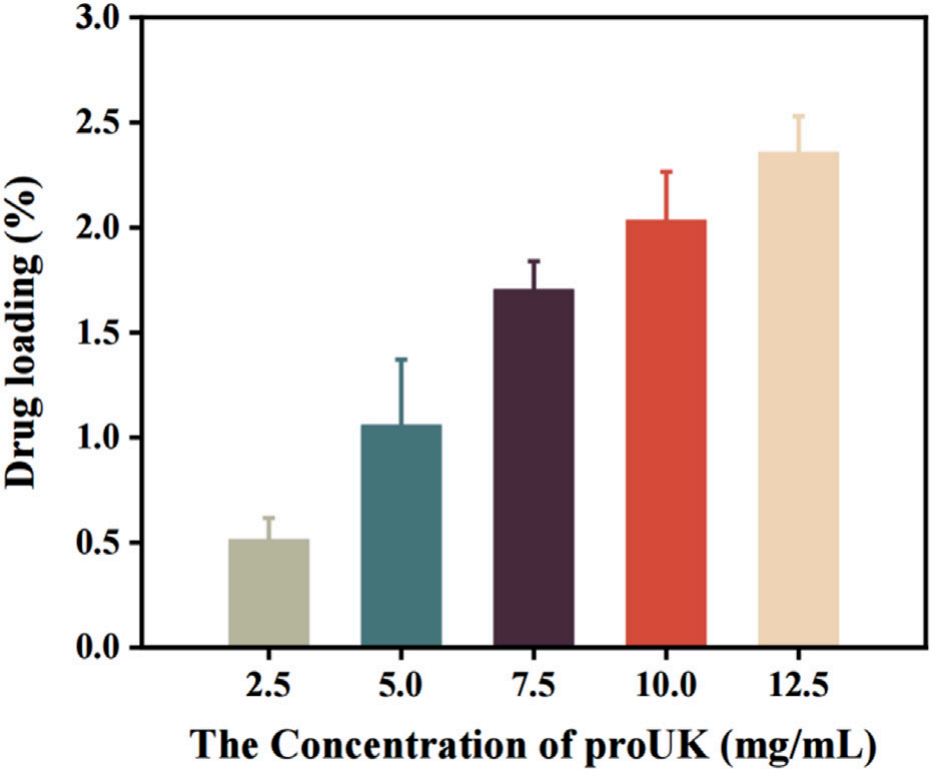
The trend of drug loading rate of microbubbles (MRB@proUK) with increasing drug concentration.

Figure 5 presents the trend in drug loading capacity of MRB@proUK microbubbles as a function of drug concentration. Preliminary analysis indicates that at low drug concentrations, the loading efficiency is relatively low (0.51%), likely because the microbubbles have not yet reached their maximal loading capacity, resulting in fewer drug molecules being encapsulated. As the drug concentration increases, a gradual rise in loading efficiency is observed, indicating enhanced drug incorporation by the microbubbles. Although the encapsulation efficiency decreases at higher drug concentrations, the overall loading rate continues to increase due to the greater total amount of drug added, and the possibility that microbubbles may adsorb more drug molecules on their surfaces. This explains why, beyond a concentration of 7.5 mg/mL, the encapsulation efficiency declines while the loading rate continues to rise.

### 3.2 The characteristics of PLGA microbubbles based on Fe_3_O_4_

**FIGURE 6:**
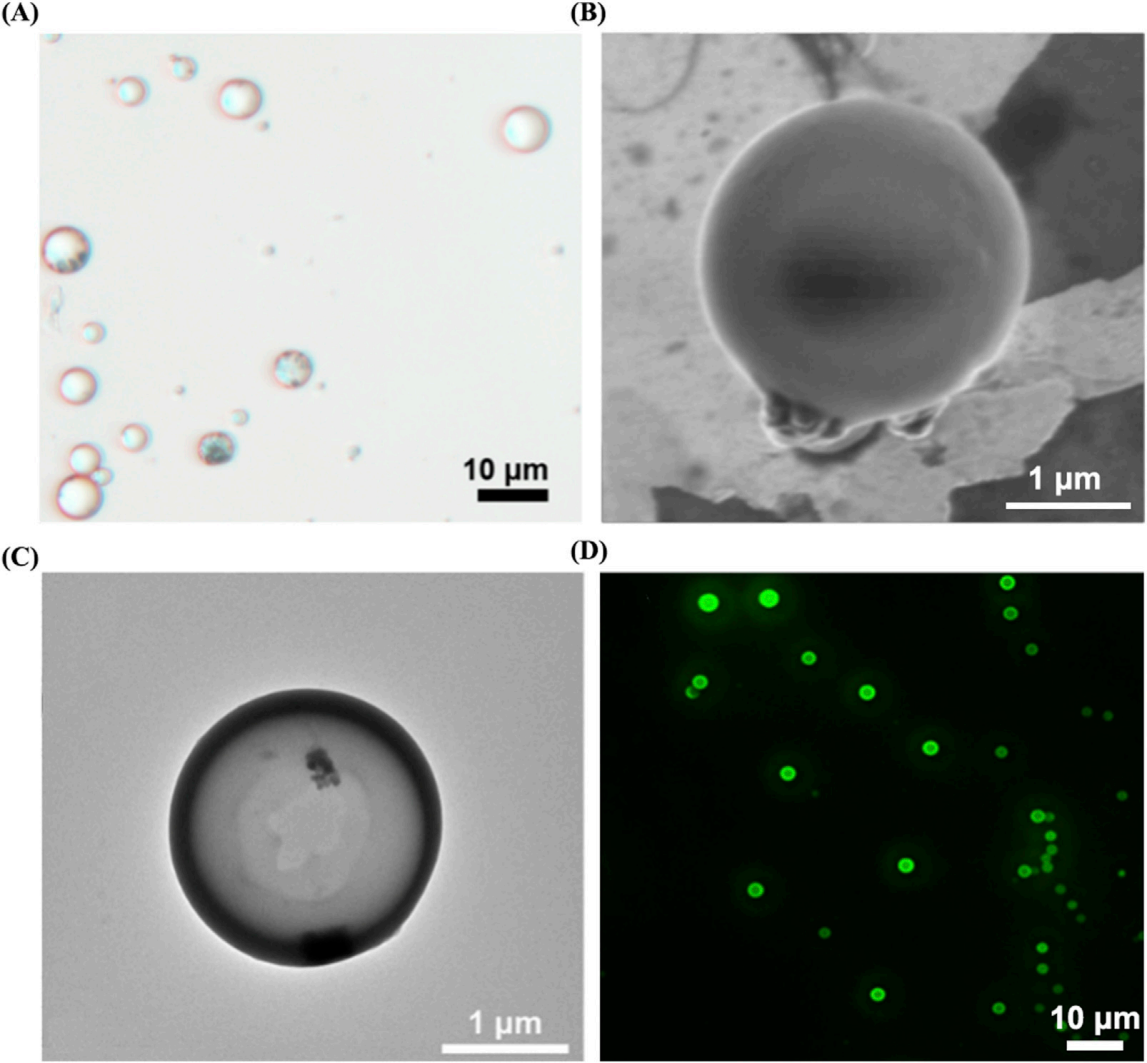
Morphological characterization of MRB@proUK microbubbles. **(A)** Under an optical microscope, the microbubble particle size was uniformly distributed at 3.7 ± 1.1. **(B)** SEM and **(C)** TEM images shown the distribution of magnetic particles in the shell of the microbubbles. **(D)** Laser scanning confocal microscopy image of MRB@proUK microbubbles, showing the microbubbles encapsulated with green fluorescence. MRB: magnetic rotation microbubbles, proUK: single-chain urokinase-type plasminogen activator, SEM: scanning electron microscope, TEM: transmission electron microscopy.

The aforementioned microbubbles were successfully prepared via the double emulsion method. Through observation under an optical microscope (as shown in Figure 6A), we found that the particle size distribution among MRBs is relatively uniform, exhibiting a spherical appearance, smooth surface, and excellent dispersibility. Observation results from scanning electron microscopy and transmission electron microscopy (as shown in Figures 6B,C) indicate that the iron oxide particles are evenly distributed within the microbubble shell. Observation using a confocal laser microscope further revealed the presence of fluorescent material inside the microbubbles (as shown in Figure 6D), confirming that the FITC-labeled proUK has been successfully loaded into the microbubbles. Detailed data regarding the average particle size, zeta potential, Fe_3_O_4_ loading rate, and proUK encapsulation efficiency of the microbubbles can be found in Table 1. It's worth noting that when the microbubbles are loaded with Fe_3_O_4_ particles, their zeta potential decreases, indicating an improvement in the dispersibility of the microbubbles. Simultaneously, we observed an increase in particle size, which may be attributed to the presence of Fe_3_O_4_ particles in the microbubble shell.

**TABLE 1:** Characteristics of the Fe_3_O_4_-Based PLGA microbubbles.

Microbubble properties	MB	MRB	MRB@proUK
Size of microbubbles (μm)	2.3 ± 0.2	3.1 ± 0.1	3.0 ± 0.3
Zeta potential (mV)	−0.4 ± 0.1	−26.0 ± 1.6	−27.4 ± 1.3
carrier rate of Fe_3_O_4_ (%)	-	45.6 ± 3.4	43.9 ± 4.3
encapsulation efficiency of proUK (%)	-	-	56.6 ± 2.2

MB, microbubbles; MRB, magnetic rotation microbubbles; MRB@proUK , magnetic rotation microbubbles with single-chain urokinase-type plasminogen activator.

### 3.3 The magnetic field properties of microbubbles

**FIGURE 7:**
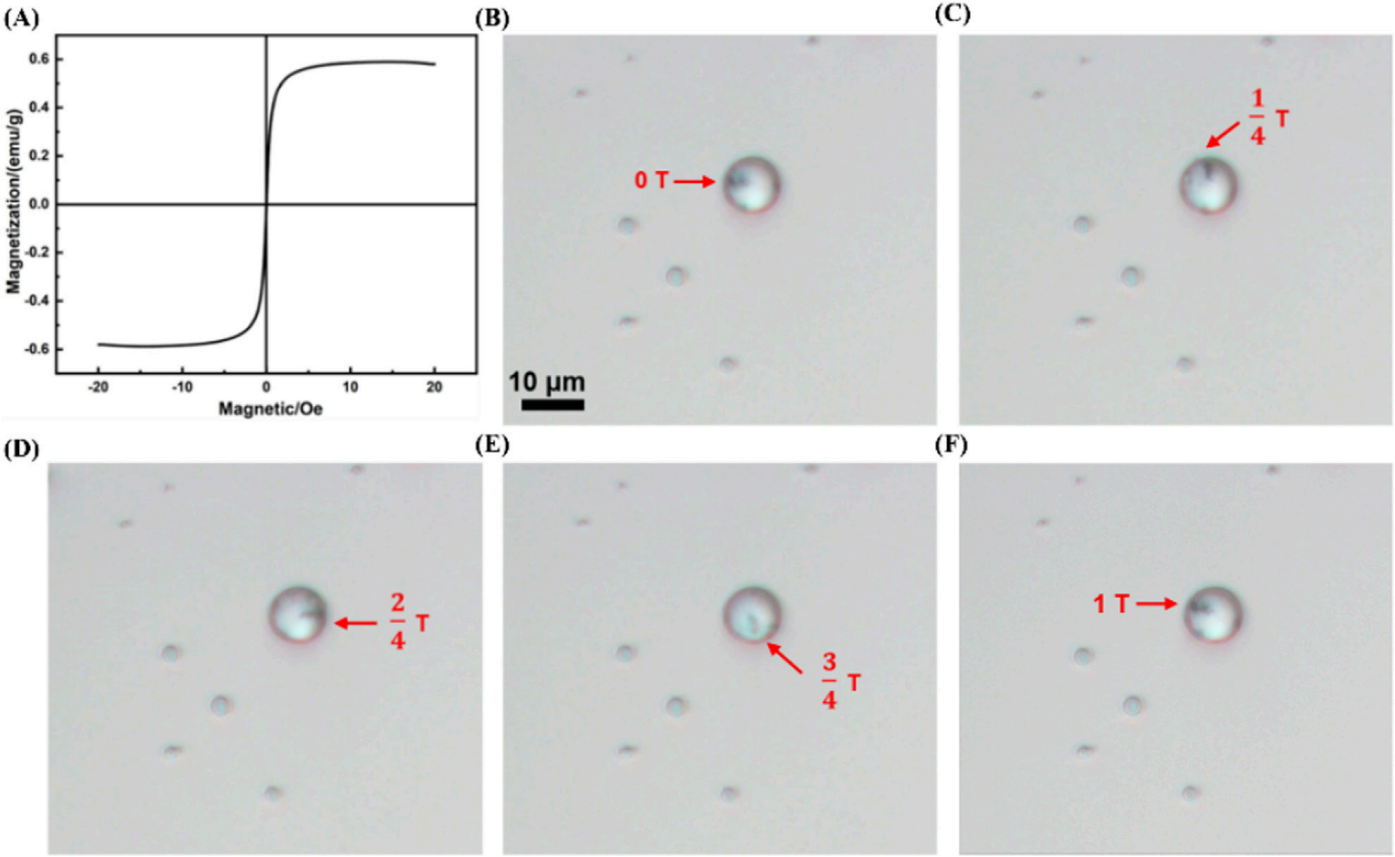
Magnetic characteristics of MRB@proUK microbubbles. **(A)** Hysteresis loop test (VSM) of MRB@proUK microbubbles. **(B–F)** Images showing the rotation of MRB@proUK microbubbles over one cycle under the influence of a rotating magnetic field (B = 2mT, f = 4 Hz). MB = microbubbles; MRB = magnetic rotation microbubbles; MRB@proUK = magnetic rotation microbubbles with single-chain urokinase-type plasminogen activator; VSM = vibrating sample magnetometer.

Additionally, under an external magnetic field of 3 mT, MRB@proUK exhibits a saturation magnetization value of 0.6 emu/g, demonstrating superparamagnetic properties (as shown in Figure 7A). Furthermore, we observed that under the influence of a rotating magnetic field (B = 2 mT, f = 4 Hz), a complete rotation cycle of the MRB@proUK microbubbles was successfully captured using an optical microscope (as shown in Figures 7B–F).

### 3.4 Research on the biocompatibility of microbubbles in vitro

**FIGURE 8:**
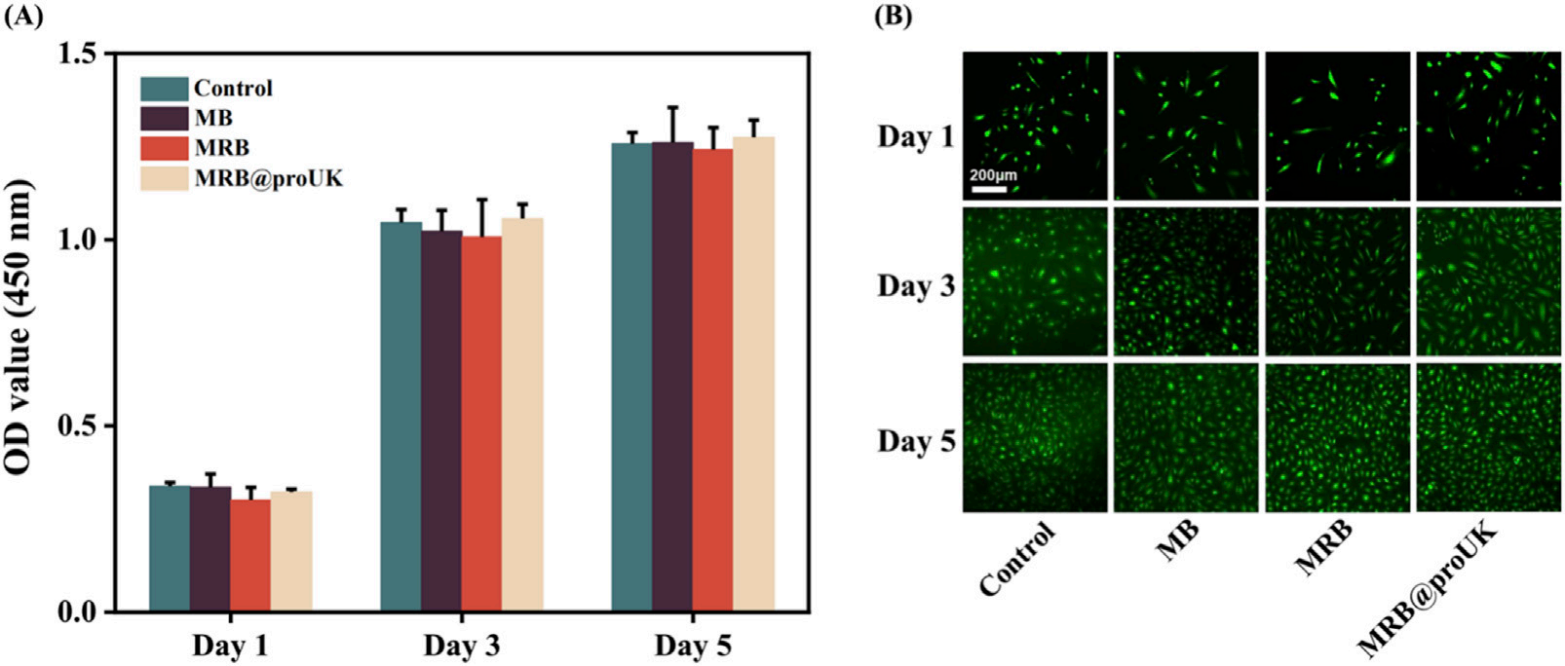
Biocompatibility experiments of microbubbles *in vitro*. **(A)** CCK8 cell proliferation assay for Control, MB, MRB, and MRB@proUK microbubbles. **(B)** Cell viability staining images of Control, MB, MRB, and MRB@proUK over 5 days. MB: microbubbles; MRB: magnetic rotation microbubbles; MRB@proUK: magnetic rotation microbubbles with single-chain urokinase-type plasminogen activator.

Figure 8A presents the results of a CCK-8 cell proliferation experiment. On the first day of the experiment, the OD values of all experimental groups were relatively low, which may be due to the fact that cells need some time to adapt to the new growth environment after inoculation. As the experiment progressed, on days 3 and 5, the OD values of the control group, MB group, MRB group, and MRB@proUK group all showed significant increases, indicating cell proliferation activity. It's worth noting that during the entire experimental period from day 1 to day 5, there were no significant differences in cell proliferation between the MB, MRB, and MRB@proUK treatment groups compared to the control group. This observation suggests that under our experimental conditions, these experimental materials did not have a significant negative impact on cell proliferation. Figure 8B sequentially presents the cell staining results of the control group, MB group, MRB group, and MRB@proUK group. Typically, live cells emit green fluorescence, while dead cells may emit fluorescence of other colors or no fluorescence at all. After careful observation of the fluorescence intensity and coverage of cells in each group, we found that the cells in all experimental groups exhibited strong green fluorescence, which strongly indicates high cell survival rates. Based on the above experimental results, we can conclude that these experimental materials exhibit low toxicity characteristics at the cellular level.

### 3.5 Influence of magnetic field strength and frequency on the thrombolysis efficiency of microbubbles

**FIGURE 9:**
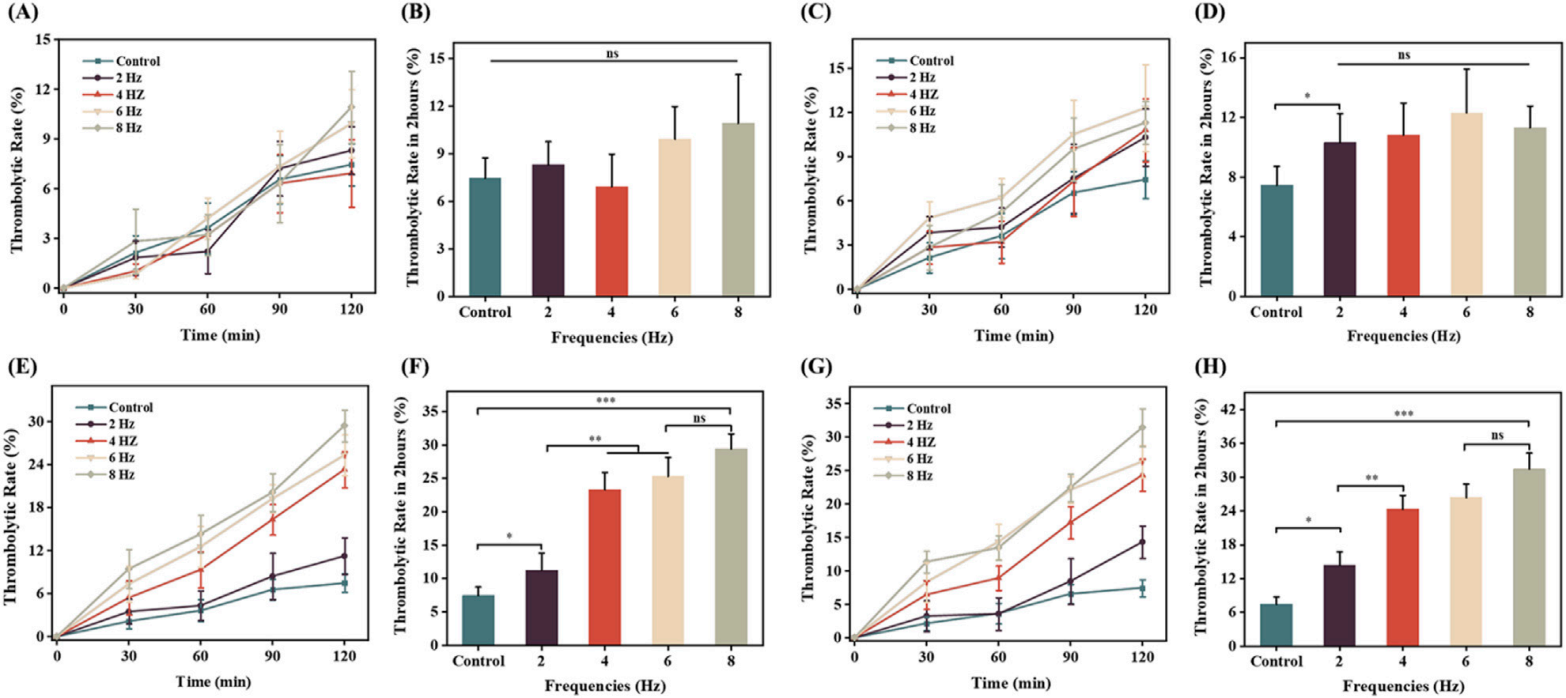
The impact of magnetic field strength and frequency on thrombolytic efficiency *in vitro*. **(A)** and **(B)** Comparison of thrombolytic rates at a rotating magnetic field strength of 0.5 mT over 2 h; **(C)** and **(D)** Comparison of thrombolytic rates at a rotating magnetic field strength of 1 mT over 2 h; **(E)** and **(F)** Comparison of thrombolytic rates at a rotating magnetic field strength of 1.5 mT over 2 h; **(G)** and **(H)** Comparison of thrombolytic rates at a rotating magnetic field strength of 2 mT over 2 h.

To further investigate the effects of combined therapy using rotating magnetic fields and magnetic microbubbles in thrombolysis treatment, we conducted a comprehensive analysis of multiple treatment cases. As shown in Figure 9A, under a magnetic field strength of 0.5 mT, even when the frequency was changed (2 Hz, 4 Hz, 6 Hz, 8 Hz), the trend of thrombolysis rate was similar to that of the control group (no magnetic field applied). This indicates that within the experimental magnetic field strength (0.5 mT) and tested frequency range, the magnetic field had no significant impact on the thrombolysis rate, showing no significant difference compared to the control group. Figure 9B further confirms this result, showing that at a magnetic field strength of 0.5 mT, different frequencies did not significantly affect the thrombolysis rate. This may be due to the fact that a smaller magnetic field strength is insufficient to provide effective vibration and rotation for the microbubbles, resulting in no significant difference in drug release efficiency compared to the control group.

As shown in Figure 9C, under a magnetic field strength of 1 mT, the thrombolysis rate of magnetically loaded microbubbles was higher compared to the blank control group, with the highest thrombolysis rate of 12% at a frequency of 6 Hz. This suggests that increasing the magnetic field strength helps improve the thrombolysis rate. Additionally, at this magnetic field strength, there was no significant difference in thrombolysis rates among different frequencies (Figure 9D), indicating that both magnetic field strength and frequency may be key factors in optimizing thrombolysis efficiency.

Figure 9E provides the experimental results of the influence of different frequencies (2 Hz, 4 Hz, 6 Hz, 8 Hz) on the thrombolysis rate under a magnetic field strength of 1.5 mT. The thrombolysis rate of the magnetic field-applied group was significantly higher than that of the blank control group, and it increased with the increase in magnetic field frequency. At a frequency of 6 Hz, the thrombolysis rate was 25%, and at 8 Hz, it reached the highest value of 29%. This may be because, as the magnetic field strength increases, higher frequency magnetic fields can more effectively stimulate the vibration and rotation of magnetic microbubbles, thereby enhancing their ability to dissolve blood clots. Figure 9F further verifies that the thrombolysis rate increases with the increase in magnetic field frequency, but the increase becomes smaller after 6 Hz, suggesting that 6 Hz is a relatively optimal frequency for thrombolysis at this magnetic field strength.

Figure 9G shown that as the magnetic field strength increased to 2 mT, the *in vitro* thrombolysis rate also increased. At a frequency of 6 Hz, Figure 9H shown the thrombolysis rate was 26%, and at 8 Hz, it reached a maximum of 31%, but the increase was small and not statistically significant. This may be due to the fact that at higher magnetic field strengths, the vibration and rotation of magnetic microbubbles reach saturation, leading to a gradual stabilization of drug release efficiency and a slower increase in the *in vitro* thrombolysis rate.

### 3.6 The level of D-dimer in the body

**FIGURE 10:**
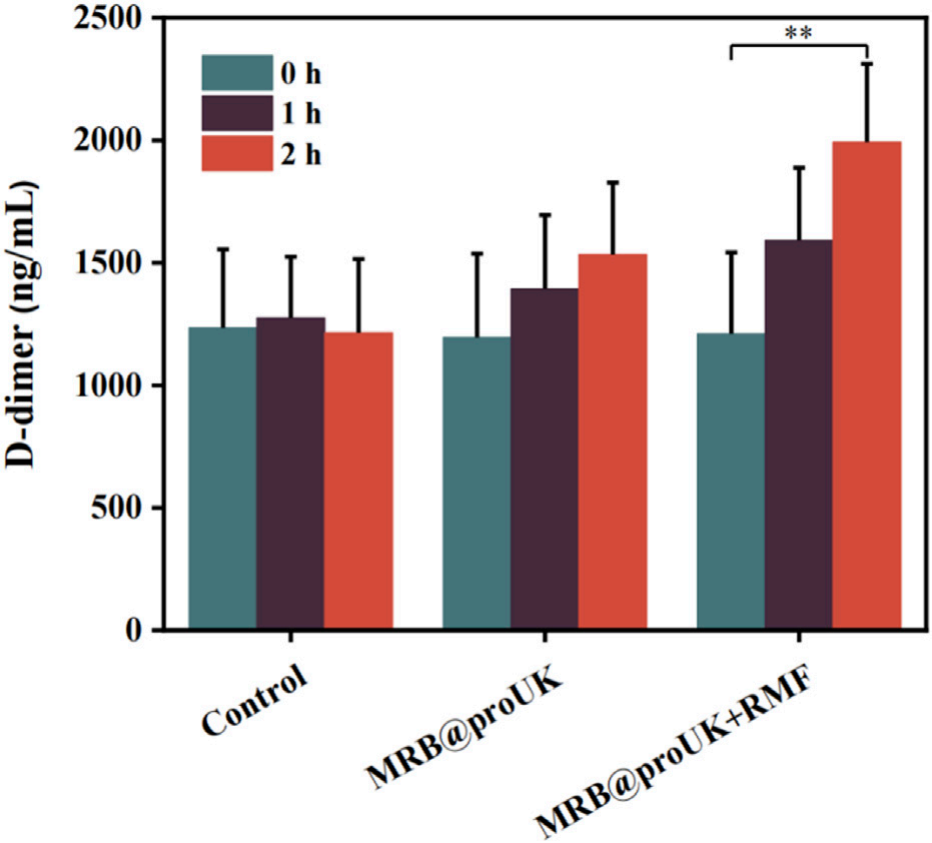
The level of D-dimer in the body.

The level of D-dimer reflects the activity of fibrinolysis in the body. As shown in Figure 10, the D-dimer levels in the blank control group were around 1200 ng/mL before and after the experiment, indicating no significant change. The D-dimer levels in the MRB group showed some fluctuation but without significant difference. In contrast, the D-dimer levels in the MRB@proUK + RMF treatment group significantly increased to approximately 2000 ng/mL. This increase indicates that a substantial amount of fibrin was degraded, leading to a rise in D-dimer levels in the blood. This indicates that the MRB@proUK + RMF treatment effectively enhanced fibrinolysis, demonstrating its potential efficacy in thrombolytic therapy.

### 3.7 Application of microbubbles in rabbit lower limb thrombosis

**FIGURE 11:**
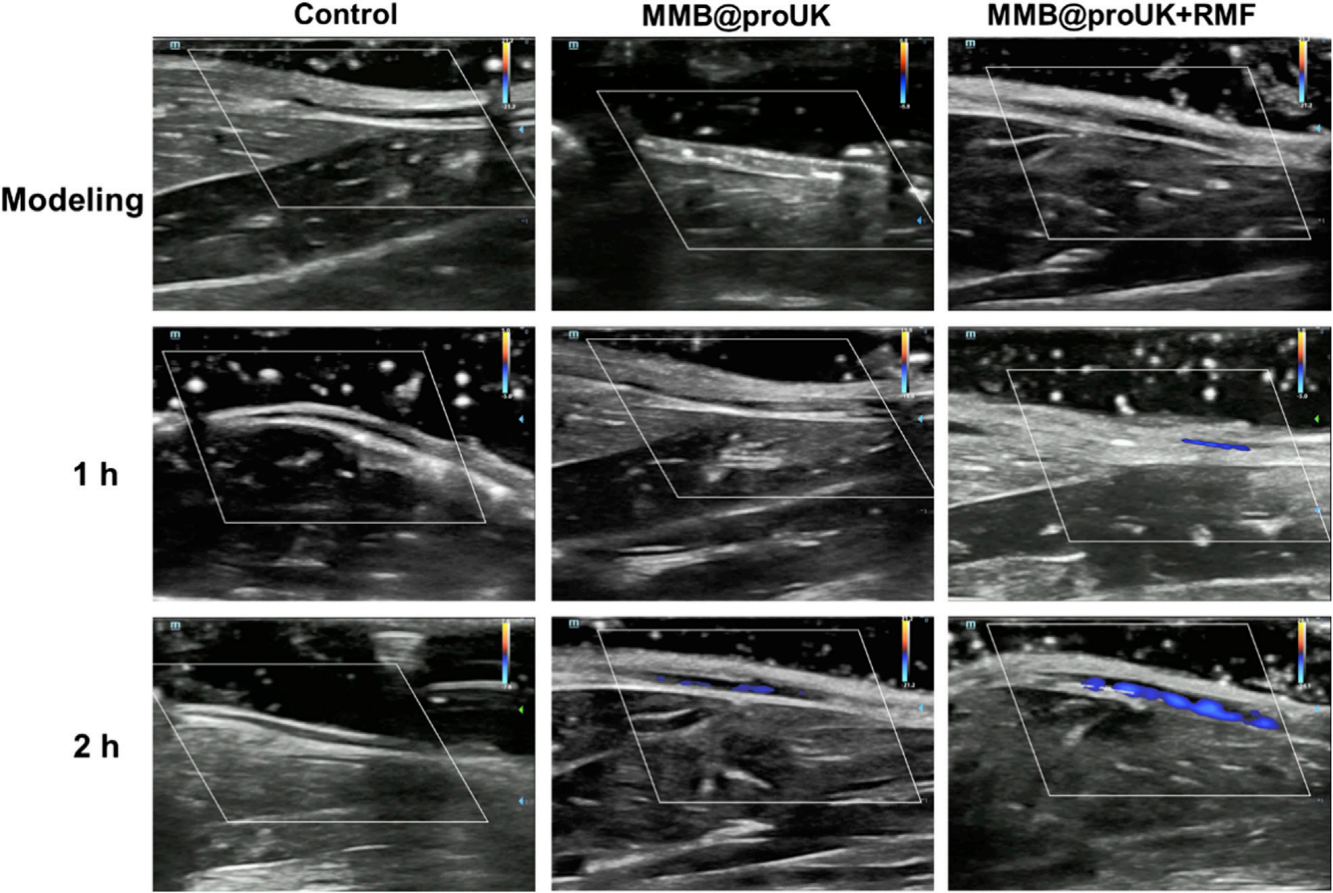
CDFI inspection image of rabbit hind limb veins.

In this study, we used rabbit hindlimb venous thrombosis as an experimental model, conducted animal thrombolysis experiments using a rotating magnetic field (RMF) set at B = 1.5 mT and f = 6 Hz. During the experiment, we conducted an in-depth analysis of the magnetic thrombolysis effect through the imaging capabilities of the ultrasound diagnostic instrument and changes in rabbit D-dimer levels. In the process of evaluating the thrombolytic effect, we utilized Color Doppler Flow Imaging (CDFI), which aids doctors in observing blood flow recovery before and after treatment, such as whether blood vessels have been reopened and whether blood flow has become smoother. For some blood vessels, if the CDFI shows obstructed blood flow before treatment, meaning no color display of blood flow signal, and the blood flow signal color significantly recovers after treatment, this change visually demonstrates the effect of thrombolytic therapy. As shown in Figure 11, in the blank control group and the experimental group using only MRB@proUK microbubbles, no blood flow signal was observed in the obstructed segment within 2 h, indicating that normal saline does not have the ability to dissolve obstructive blood clots, and the clots are not easily detached from the blood vessel walls. However, in the experimental group with the application of a magnetic field (MRB@proUK + RMF), a clear blue blood flow signal can be observed after 2 h, which clearly indicates that the blood clot has been successfully dissolved.

D-dimer is a special protein fragment produced during the process of thrombus formation and dissolution. It is a product of fibrin decomposition and is often used as a biomarker to detect thrombosis and fibrinolytic activity in the body. The level of D-dimer reflects the activity of fibrinolysis in the body. The D-dimer level in the blank control group remained at around 1,200 ng/mL before and after the experiment, with basically no change. Although there were fluctuations in the D-dimer level in the MRB group, there was no significant difference. The D-dimer level in the MRB@proUK + RMF group increased significantly after treatment, reaching around 2,000 ng/mL. This indicates that a large amount of fibrin was decomposed. Therefore, the level of D-dimer in the blood increased.

### 3.8 Histopathological analysis of treated veins

**FIGURE 12:**
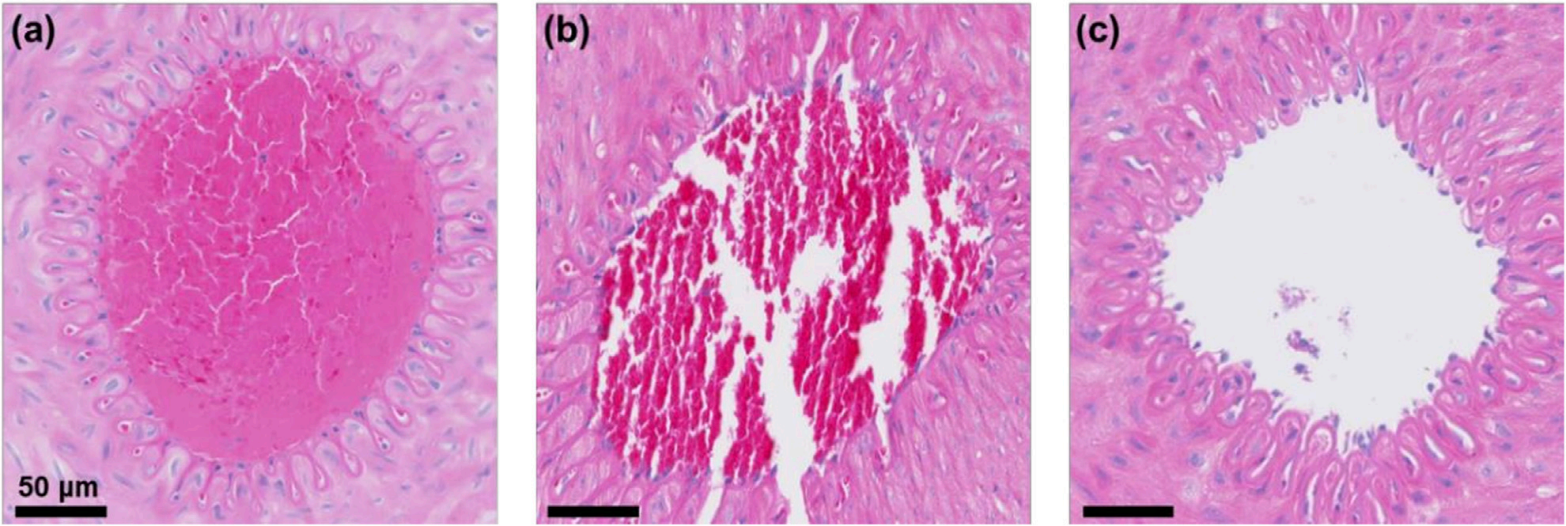
Vascular H&E staining results. **(a)** Blank control group; **(b)** MRB@proUK group; **(c)** MRB@proUK + RMF group.

**FIGURE 13:**
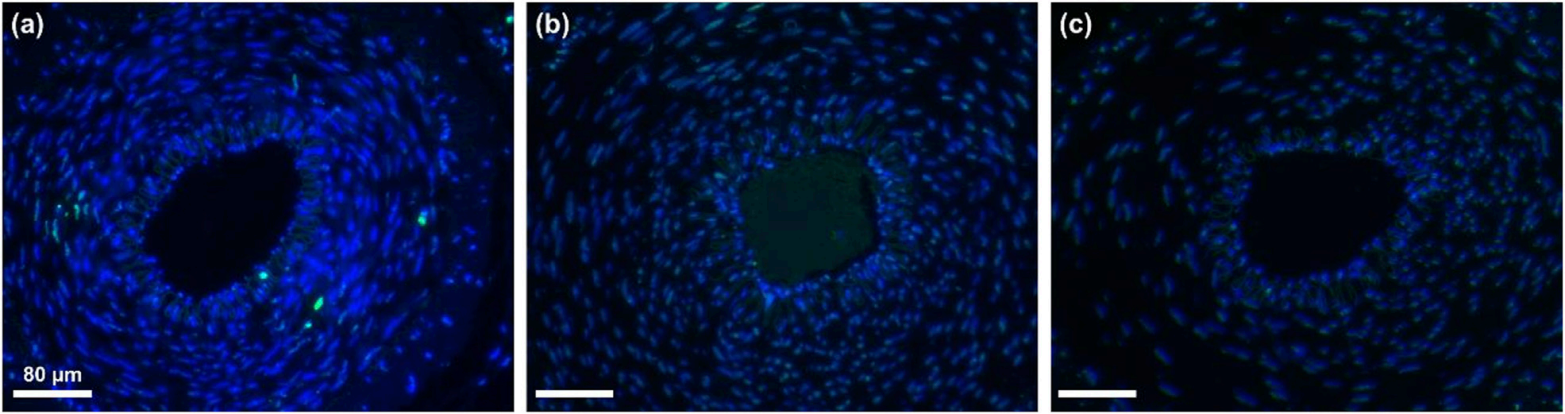
Results of vascular TUNEL fluorescence staining **(a)** Blank control group **(b)** MRB@proUK group **(c)** MRB@proUK+RMF group.

To verify whether the low-frequency rotating magnetic field (RMF) causes vascular damage, the treated vessel segments were harvested and subjected to H&E staining and TUNEL immunofluorescence staining. DAPI was used to stain cell nuclei, producing a blue fluorescence, while FITC labeled DNA fragmentation, causing apoptotic cells to emit green fluorescence. As shown in Figure 12, the MRB@proUK + RMF group exhibited clearly defined cell nuclei and endothelial cell morphology similar to that of the blank control group, with no obvious vascular damage observed. In the RMF-treated group (Figure 12c), the thrombus was almost completely dissolved, and the vessel lumen was patent. In contrast, the blank control group and the MRB@proUK group showed varying degrees of partial thrombus dissolution. As presented in Figure 13, TUNEL fuorescence staining revealed varying intensities of blue and green fuorescence in all three groups. However, the intensity of blue fuorescence was significantly higher than that of green fluorescence, indicating that although some degree of apoptosis was present across the groups, it remained within the normal physiological range (Figures 13a,b). These results further confrm that the RMF treatment does not cause significant damage to the vascular tissue (Figure 13c).

## 4 Discussion

Thrombosis is the main pathological mechanism leading to acute ischemic cardiovascular diseases, posing a serious threat to patients’ health and life. Currently, although there are various commonly used thrombolytic therapies, such as pharmacologic thrombolysis, surgical thrombectomy, and ultrasound thrombolysis, these treatment methods can sometimes cause serious or even fatal complications (Bai et al., 2024). In contrast, magnetic field-mediated treatment strategies exhibit unique advantages. It utilizes rotating magnetic fields to manipulate magnetic microbubbles for rotation and perturbation in the target thrombus area, thereby promoting drug release and achieving treatment for acute venous thrombosis (Friedrich et al., 2021). It's worth noting that magnetic fields can safely penetrate human tissues, including the head, under low-frequency conditions without causing damage to biological tissues (Adair, 2000). Additionally, the operation of the magnetic field has time selectivity and spatial limitation, which benefits from the particle-mediated interaction mechanism (Cheng et al., 2016). The magnetic microbubbles we developed have magnetic particles embedded in their shell layers, enabling rotational movement under the influence of a magnetic field. Experimental results show that the magnetic microbubble material exhibits non-toxic behavior towards cells, meeting the standards for biomedical materials.

We developed magnetic microbubbles with superparamagnetic iron oxide nanoparticles embedded in their shell layer. This approach offers several advantages: Firstly, studies have shown that iron oxide nanoparticles are biocompatible and degradable within the body (Weissleder et al., 1989; Pouliquen et al., 1991). Secondly, drug controlled release can be achieved by altering the strength and frequency of the magnetic field (Marín et al., 2018; Kang et al., 2022). In this study, we measured the OD values through cell proliferation experiments and found no statistically significant difference between the control group and the experimental group, indicating that the microbubbles did not inhibit cell proliferation. Additionally, cell viability assays and live/dead cell staining images showed no observed cell death, suggesting that these magnetic microbubbles exhibit no significant cytotoxicity, meeting the requirements for use as biomedical materials. Furthermore, we observed that under a rotating magnetic field of 1.5 mT and 6 Hz frequency, the thrombolysis rate of the microbubbles reached 25%, showing a significant increase compared to the control group, demonstrating the feasibility of controlling drug release from magnetic microbubbles using alternating magnetic fields. However, our research also found that at higher magnetic field strengths (such as 2 mT), the improvement in thrombolysis efficiency became less pronounced, possibly due to the saturation of vibration and rotation of the microbubbles.

It is noteworthy that although our study demonstrated promising preliminary results, several challenges must be overcome before clinical application. For instance, this study did not consider the issue of heat generation by magnetic nanoparticles. Under the influence of an alternating magnetic field, magnetic nanoparticles produce and release heat into the environment (Rosensweig, 2002; Jordan et al., 1999). Unselective continuous exposure of magnetic nanoparticles within the body to alternating or high-frequency magnetic fields could damage healthy tissues (Thiesen and Jordan, 2008). While the rotating magnetic field used in the study (0–2 mT strength, 0–8 Hz frequency) is considered safe for humans and does not cause damage to surrounding tissues, practical clinical applications require that the magnetic field-generating equipment operate stably in a hospital setting and be adjustable according to individual patient conditions.

In addition, ensuring precise delivery of the magnetic microbubbles to the target site remains a key challenge. Although ultrasound-guided administration can increase local microbubble concentration, achieving accurate spatial targeting is still difficult (Wu et al., 2025). Meanwhile, although preliminary cytotoxicity and animal studies suggest that the materials used are biocompatible, further data are needed to evaluate their long-term safety and suitability for use in larger patient populations. Moreover, the current rotating magnetic field generation devices are relatively bulky, which poses practical limitations for clinical deployment—particularly in emergency situations. Therefore, the development of more compact and portable magnetic field generators is essential. In summary, in order to achieve successful clinical translation, it is necessary not only to address the issues of device miniaturization and portability but also to improve the targeting accuracy of the drug delivery system and ensure long-term safety. Only by overcoming these barriers can this technology make the critical transition from bench to bedside.

Srinivas et al. (2014), Minh et al. (2021), Xue et al. (2025), Siciliano et al. (2024) found that, catheter guided thrombolysis (CDT) and assisted mechanical thrombolysis are now considered the medical standards for deep vein thrombosis (DVT). This study aims to describe the immediate and long-term (6 months) safety and efficacy of CDT in patients with lower limb DVT compared to conventional anticoagulant therapy alone. All patients aged 12 to 85 who have recently (0–8 weeks) developed DVT were included in the study. The CDT group mechanically aspirated blood clots and was given streptokinase (STK) and unfractionated heparin (UFH) simultaneously. Six months later, dual power ultrasound and Villalta scale were used to evaluate deep vein patency and post thrombotic syndrome (PTS). Among the 51 patients with complete data, 25 patients were assigned additional CDT with an average duration of 108 days ±32.26 patients received standard treatment only. 37% of patients achieved grade III (complete) dissolution, and 63% of patients achieved grade II (50%–90%) dissolution. Partially dissolved patients underwent percutaneous transluminal angioplasty and/or venous stent implantation. After 6 months, the iliofemoral artery patency rate in the CDT group was 20 (80%), while in the anticoagulation group alone it was 7 (23%) (p < 0.01) In the CDT group, 5 cases (20%) developed PTS, while in the anticoagulation group alone, 19 cases (77%) developed PTS (p < 0.01) We conclude that CDT and conventional manual thrombectomy are effective methods for treating deep vein thrombosis in the lower limbs. STK infusion can be safely administered for up to 6 days. Due to the fact that adding UFH can cause thrombocytopenia, daily monitoring of whole blood cell count is required during CDT.

In conclusion, this study developed a PLGA-based magnetic drug-loaded microbubble aimed at enhancing the dissolution of acute lower limb venous thrombosis via controllable rotating magnetic fields. Our experimental results show that this novel magnetic drug delivery system exhibits significant thrombolysis effects both *in vitro* and *in vivo* models. By combining magnetic microbubbles with rotating magnetic fields, we provide a safe and effective treatment strategy for acute venous thrombosis. Future work should focus on further optimizing this system and exploring its potential in treating a broader range of diseases.

## Data Availability

The original contributions presented in the study are included in the article/Supplementary Material, further inquiries can be directed to the corresponding authors.
