# Correction: Magnetic drug-loaded microbubbles for treating lower limb venous thrombosis under controllable rotating magnetic field

**DOI:** 10.3389/fbioe.2025.1755810

**Published:** 2025-12-04

**Authors:** Frontiers Production Office

**Affiliations:** Frontiers Media SA, Lausanne, Switzerland

**Keywords:** poly (lactic-co-glycolic acid) microbubbles, magnetic drug delivery, rotating magnetic field, thrombolysis, single-chain urokinase-type plasminogen activator (proUK), acute lower limb venous thrombosis

**Author** Yu Zhang was erroneously included as an author in the published article. The correct **Author list** reads:

Yu-Ming Huang1,2†, Cheng-Rang Liu3†, Yi-Qi Xu4†, Chao Cao3†, Zhen-Gan Huang5, Hong-Wen Fei5* and Yue-Shan Huang3*

1School of Medicine, South China University of Technology, Guangzhou, China, 2Department of Catheterization Lab, Guangdong Cardiovascular Institute, Guangdong Provincial Key Laboratory of South China Structural Heart Disease, Guangdong Provincial People’s Hospital (Guangdong Academy of Medical Sciences), Southern Medical University, Guangzhou, China, 3School of Materials Science and Engineering, South China University of Technology, Guangzhou, Guangdong, China, 4Department of Pediatrics, Nanfang Hospital, Southern Medical University, Guangzhou, China, 5Guangdong Cardiovascular Institute, Guangdong Provincial People’s Hospital (Guangdong Academy of Medical Sciences), Southern Medical University, Guangzhou, China

The **Author Contributions Statement** has been corrected to read:

Y-MH: Writing – original draft, Writing – review and editing. C-RL: Writing – original draft, Writing – review and editing. Y-QX: Writing – original draft, Writing – review and editing. CC: Writing – review and editing, Writing – original draft. Z-GH: Writing – review and editing. H-WF: Writing – review and editing. Y-SH: Writing – review and editing.

The original article has been updated.

