# Supplementary figures and images for: Magnetic drug-loaded microbubbles for treating lower limb venous thrombosis under controllable rotating magnetic field
Source: Front Bioeng Biotechnol. 2025 Nov 12;13:1615863. doi: 10.3389/fbioe.2025.1615863 (PMC12650771; doi:10.3389/fbioe.2025.1615863)

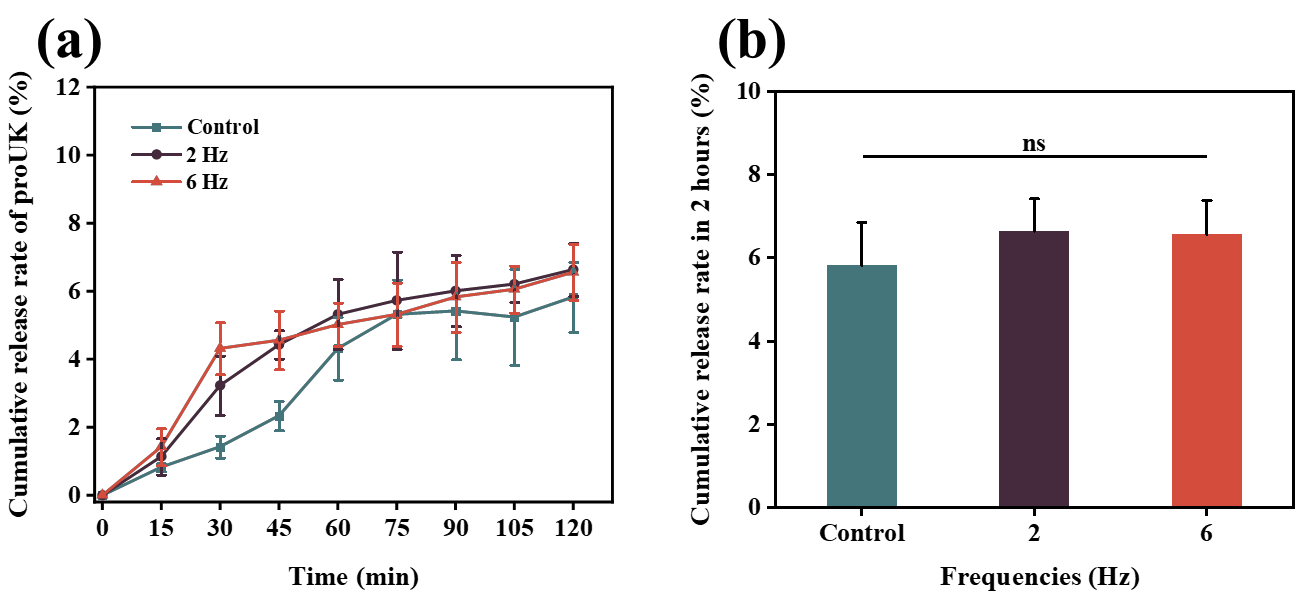

Supplement: Supplementary file 1 [file Image2.tif]

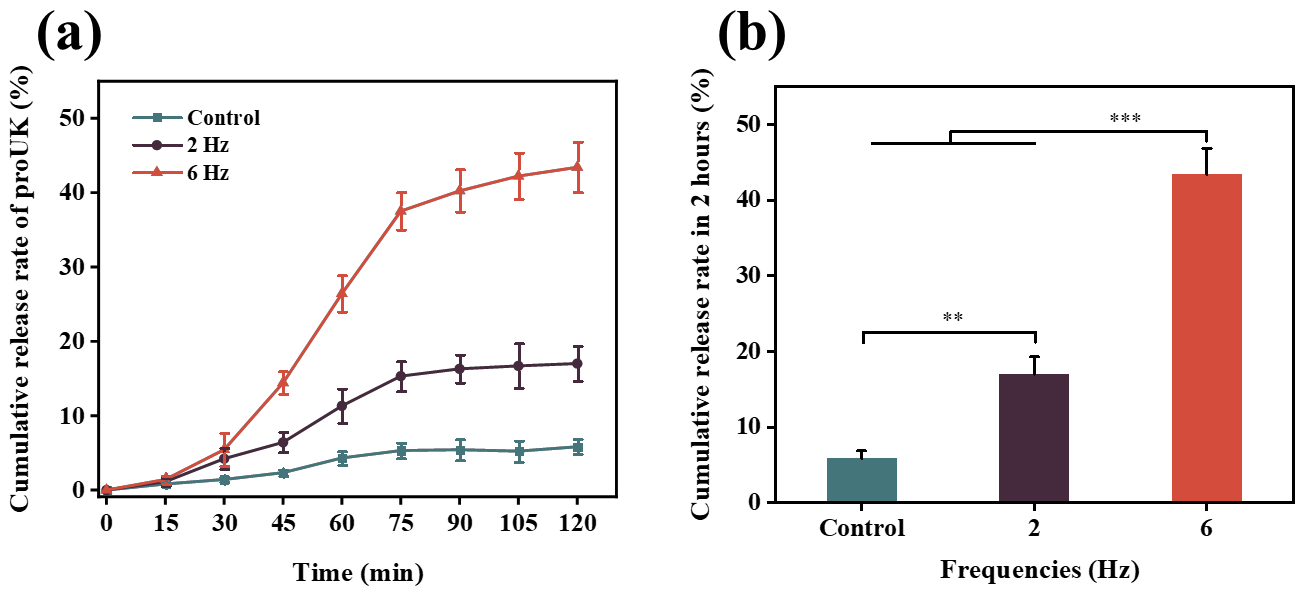

Supplement: Supplementary file 2 [file Image1.tif]
